# Coordinated motions of multiple robotic manipulators with matrix-weighted network

**DOI:** 10.1038/s41598-022-15939-4

**Published:** 2022-07-12

**Authors:** Liyun Zhao, Yan Ren, Rui Wang

**Affiliations:** 1 School of Science, Inner Mongolia University of Science and Technology, Baotou, 014010 People’s Republic of China; 2 School of Information Engineering, Inner Mongolia University of Science and Technology, Baotou, 014010 People’s Republic of China; 3 School of Applied Mathematics, Shanxi University of Finance and Economics, Taiyuan, 030012 People’s Republic of China

**Keywords:** Engineering, Mathematics and computing, Physics

## Abstract

This paper addresses coordinated problem of uncertain robotic manipulators with matrix-weighted network. Given interconnections between agents are weighted by nonnegative definite matrices, we present a sufficient and necessary condition about zero eigenvalues of matrix-weighted Laplacian and types of coordinated behaviors for multiple agents. Based on the condition, two novel control schemes are proposed for the networked robots by introducing matrix-weighted network. We employ the decomposition approach and Lyapunov-like approach to show coordinated motions of the networked system, and demonstrate that the proposed controls are capable of ensuring the robotic agents reach complete/cluster consensus and complete/cluster synchronization. Finally, some numerical examples and simulations demonstrate the obtained theoretical results.

## Introduction

In the last decade, much effort has been devoted to the cooperative control problem for a group of dynamic agents because of its widespread applications in a variety of fields^1–3^. As a consequence, up to now, a large quantity of coordinated control protocols (or algorithms) have been presented and all kinds of cooperative motions, like consensus^3^, synchronization^4^, tracking^5^, cluster^6^, flocking^7^, formation^8–11^, have been investigated. It should be noted that the above-mentioned works were mainly focused on the scalar-weighted interactions among agents, which is not applicable in some practical scenarios, especially when interdependencies among high-dimensional states of neighboring agents are discussed^12,13^. For example, as we all know, the exchanges of opinions between people are always sophisticated, in particular, when multiple relevant topics are considered. Besides interaction between members in the social network, one man can perceive another from different topics. Thereby, a positive semidefinite matrix naturally is assigned to characterize such elaborate and topic-dependent interaction^14,15^. In other areas, matrix-weights are also introduced, such as matrix-weighted consensus of multiple first-order (second-order) integrators^15–17^, bipartite consensus of matrix-weighted networked first-order integrators^14,18^, synchronization of coupled-oscillator networks^19^, the controllability of matrix-weighted networks^20^ and so on.

The aforementioned literatures on matrix-weighted consensus concentrate almost on the linear multiple agent systems. However, the results are inapplicable for many real applications, such as formation of multiple spacecraft, cooperation of multiple nonlinear robotic manipulators^21^. These plants are usually known as mechanical systems, which can be described as Euler-Lagrange (EL) dynamics^22–24^. Because of intrinsic strong nonlinearity of robotic manipulator systems and complexity of matrix-weighted Laplacian, coordinated control of robotic manipulator systems with matrix-weighted Laplacian is more challenging. As a result, to our knowledge, there are almost no the related researches. Different from scalar-weighted networks, even when the networked topologies are connected, the clustering phenomenon naturally exists, which implies properties of matrix-weighted Laplacian are instrumental in coordination of multiple agents. Therefore, although there are many coordinated control approaches on uncertain robotic manipulators with scalar-weighted network in the existing works^25–27^, what coordinated control algorithms should be proposed and which type of coordinated behaviors will be achieved are still difficult to handle for matrix-weighted coupled robotic manipulators.

Motivated by the above discussions, this paper considers coordinated motions of uncertain robotic manipulators with matrix-weighted network. Given interconnections between agents are weighted by nonnegative definite matrices, it is shown that coordinated behaviors of multiple agents are related with the number of zero eigenvalues for Laplacian matrix. The novelties and contributions of this article are summarized as the following aspects.

(i) A sufficient and necessary condition is presented, which is about the relation between the number of zero eigenvalues for matrix-weighted Laplacian and types of coordinated behaviors for multiple agents.
(ii) Different from scalar weight in Refs.^24,28–30^, matrix-weighted network is introduced. To the end, multiple uncertain robotic manipulators reach the static and stationary cooperative motions.
(iii) Due to introduction of matrix-weighted network, the related results on scalar-weighted networked robotic manipulators^22–24^ are not available. Therefore, two novel control algorithms and some coordinated criteria are proposed such that uncertain robotic manipulators can reach complete/cluster consensus and complete/cluster synchronization.

## Preliminaries and problem formulation

In this section, we will introduce some basic preliminaries of the algebraic graph with matrix-weights, robotic manipulator and some definitions on coordinated motions of multiple agents.

### Notations

Henceforth, let $$R^{d}$$ and $$R^{n\times n}$$ be sets of *d* dimension column vectors and $$n\times n$$ matrices. The symbol $${\mathbf {0}}\in R^d$$ denotes a vector with all entries being 0. $$D=\mathrm {blkdiag}(D_1,D_2,$$ $$\ldots ,$$ $$D_n)$$ is a block diagonal matrix with diagonal blocks $$D_i(i=1,2,\ldots ,n)$$. $$A\otimes B$$ means the Kronecker product of the matrices *A*, *B*. $${\mathcal {N}}(L)$$ is the nullspace of the matrix *L*. We write $$M\succ 0 (M \succeq 0)$$ to mean *M* is a positive (semi) definite matrix. $$I_n=({\mathbf {e}}_1,{\mathbf {e}}_2,\ldots ,{\mathbf {e}}_n )\in R^{n\times n}$$ is the *n* order identity matrix, where the *i*th entry of the identity vector $${\mathbf {e}}_i(i=1,2,\ldots ,n)$$ is one and the others are zeroes.

### Matrix-weighted graph theory

Let a triple ${\mathcal {G}}=({\mathcal {V}},{\mathcal {E}},A)$ be a undirected graph composed of *m* agents, where ${\mathcal {V}}=\{1,2, \ldots ,m\}$, ${\mathcal {E}}\subseteq {\mathcal {V}}\times {\mathcal {V}}$ respectively are the sets of vertices, edges, and the matrix-weighted adjacency matrix $A=(A_{ij})_{md\times md}$. The *ij*th sub-block $A_{ij}\in R^{d\times d}$ of the block matrix *A* satisfies that $A_{ij}=A_{ij}^T$ and $A_{ij}=A_{ji}$. $A_{ij}\succ 0$ or $A_{ij}\succeq 0$ if and only if there is a edge (*i*, *j*) in ${\mathcal {G}}$, $A_{ij}= {O}$ otherwise. If $A_{ij}\succ 0(A_{ij}\succeq 0)$, the edge (*i*, *j*) is called as positive definite (positive semi-definite) edge. It is clear that the matrix-weighted adjacency matrix *A* degenerates into a usual scalar-weighted adjacency matrix when $d=1$. The block diagonal matrix $D=\mathrm {blkdiag}(D_1,D_2,\ldots ,D_m)$ with diagonal block $D_i=\sum \limits \limits_{j=1}^{m}A_{ij}$. The block matrix *L* is a Laplacian matrix, which is defined as $L=D-A$. A path between *i* and *j* is a sequence of edges of the form $(i,i_1),(i_1,i_2),\ldots ,(i_k,j)$, where each edge is positive semi-definite or positive definite. When every edge is positive definite, the path is called as positive path. A positive spanning tree is a positive tree containing all vertices in ${\mathcal {G}}$^31^. A partition $\{{\mathcal {P}}_{1}, {\mathcal {P}}_{2},\ldots , {\mathcal {P}}_{q}\}$ is a disjoint division of ${\mathcal {V}}$ satisfying ${\mathcal {P}}_{i}\bigcap {\mathcal {P}}_{j}=\emptyset (i\ne j)$ and $\bigcup \limits _{i=1}^q {\mathcal {P}}_{i}={\mathcal {V}}$^30^.

### Robotic manipulator systems

In this paper, we will consider *m* uncertain robotic manipulators, which are labelled from manipulator 1 to *m*. According to Ref.^32^, the *i*th robotic manipulator can be described by EL equation as follows:

1 $$\begin{aligned} M_i(q_i)\ddot{q}_i+C_i(q_i,{\dot{q}}_i){\dot{q}}_i+g_i(q_i)=\tau _i,~~~~i=1,2,\ldots ,m, \end{aligned}$$

where $q_i\in R^d$ denotes generalized position of manipulator *i*, $M_i(q_i)\in R^{d\times d}$ and $C_i(q_i,{\dot{q}}_i)\in R^{d\times d}$ respectively are symmetric positive-definite inertia matrix and Coriolis/centripetal matrix, $g_i(q_i)$ and $\tau _i\in R^d$ are gravitational force and the control input of the *i*th manipulator, respectively. It is well-known that there are three properties related to the Lagrangian system (1), which are listed below^33^.

*Property 1:* Symmetric positive-definite inertia matrix $M_i(q_i)$ is uniformly bounded and $\parallel C_i(q_i,{\dot{q}}_i)\parallel \le \parallel {\dot{q}}_i\parallel$.

*Property 2:* The matrix ${\dot{M}}_i(q_i)-2C_i(q_i,{\dot{q}}_i)$ is skew symmetric, i.e., for any vector $x \in R^d$, there is $x^T({\dot{M}}_i(q_i)-2C_i(q_i,{\dot{q}}_i))x={\mathbf {0}}$.

*Property 3:* For any vectors $x, y\in R^d$, there is $M_i(q_i)x+C_i(q_i,{\dot{q}}_i)y+g_i(q_i)=Y_i(q_i,{\dot{q}}_i,x,y)\varTheta _i$ with $Y_i(q_i,{\dot{q}}_i,x,y)$ being the regressor matrix and $\varTheta _i$ being a constant parameter vector.

For the above coupled manipulator system (1), we will investigate its coordinated motion, where specific coordinated motions can be defined as:

#### **Definition 1**

EL system (1) reach complete consensus if $\lim \limits _{t\rightarrow +\infty }||q_i(t)-q_j(t)||=0$ and $\lim \limits _{t\rightarrow +\infty }||{\dot{q}}_i(t)||=0$ for any $i,j\in {\mathcal {V}}$ and any initial values. If the node set ${\mathcal {V}}$ can be divided into $\{{\mathcal {P}}_{1}, {\mathcal {P}}_{2},\ldots , {\mathcal {P}}_{q}\}$ such that the following conditions are satisfied

2 $$\begin{aligned} \left\{ \begin{array}{l} \lim \limits _{t\rightarrow +\infty }||q_i(t)-q_j(t)||=0, \forall i,j\in {\mathcal {P}}_{k},\\ \lim \limits _{t\rightarrow +\infty }||q_i(t)-q_j(t)||\ne 0, \forall i\in {\mathcal {P}}_{k}, j\in {\mathcal {P}}_{l}~(k\ne l),\\ \lim \limits _{t\rightarrow +\infty }||{\dot{q}}_i(t)||=0, \forall i\in {\mathcal {V}},\\ \end{array} \right. \end{aligned}$$

we say EL system (1) achieve cluster consensus.

#### **Definition 2**

EL system (1) is said to realize complete synchronization if

3 $$\begin{aligned} \left\{ \begin{array}{l} \lim \limits _{t\rightarrow +\infty }||q_i(t)-q_j(t)||=0, \lim \limits _{t\rightarrow +\infty }||{\dot{q}}_i(t)-{\dot{q}}_j(t)||=0,\forall i,j\in {\mathcal {V}} \\ \lim \limits _{t\rightarrow +\infty }||{\dot{q}}_i(t)||\ne 0, {\forall i\in {\mathcal {V}}.} \end{array} \right. \end{aligned}$$

Suppose that

4 $$\begin{aligned} \left\{ \begin{array}{l} \lim \limits _{t\rightarrow +\infty }||q_i(t)-q_j(t)||=0, \lim \limits _{t\rightarrow +\infty }||{\dot{q}}_i(t)-{\dot{q}}_j(t)||=0, \forall i,j\in {\mathcal {P}}_{k},\\ \lim \limits _{t\rightarrow +\infty }||q_i(t)-q_j(t)||\ne 0, \lim \limits _{t\rightarrow +\infty }||{\dot{q}}_i(t)-{\dot{q}}_j(t)||\ne 0, \forall i\in {\mathcal {P}}_{k}, j\in {\mathcal {P}}_{l}~(k\ne l),\\ \lim \limits _{t\rightarrow +\infty }||{\dot{q}}_i(t)||\ne 0, {\forall i\in {\mathcal {V}},} \end{array} \right. \end{aligned}$$

we say EL system (1) admit cluster synchronization.

#### *Remark 1*

Consensus and synchronization in the above definitions are in accord with consensus and tracking synchronization in^24^, respectively. If networked robotic manipulator (1) achieves complete consensus or cluster consensus, we think it realizes stationary coordinated motion, while if complete or cluster synchronization is reached for EL system (1), we say the system achieves dynamic coordinated motion.

#### *Remark 2*

There are more restrictions in the above cluster consensus (synchronization) than group consensus in^34^. Roughly speaking, cluster consensus (synchronization) in the above definitions is in accordance with one in^35^, where consensus (synchronization) between different partitioned subgroups can’t occur, while in a group consensus in^34^, there can happen a consensus (synchronization) between two distinct partitioned subgroups.

## Coordinated motion of robotic manipulators with matrix-weighted network

### A useful lemma

In this section, we will introduce a useful lemma on coupled single-integrator system, where the *i*th single-integrator agent in the system updates its states under the following protocol

5 $$\begin{aligned} {\dot{x}}_i=-\sum \limits \limits _{j=1}^{m}A_{ij}(x_i-x_j) \end{aligned}$$

with $x_i\in R^d$ being the state of agent $i (i=1,2,\ldots ,m)$ at time instance *t*.

Based on matrix theories, a algebraic criterion will be established for networked single-integrator system to achieve complete consensus and cluster consensus, where complete consensus means $\lim \limits _{t\rightarrow +\infty }||x_i -x_j||=0$, for $i,j=1,2,\ldots ,m$ and $i\ne j$. However, if there exists a partition $\{{\mathcal {P}}_{1}, {\mathcal {P}}_{2},\ldots , {\mathcal {P}}_{q}\}$ such that $\lim \limits _{t\rightarrow +\infty }||x_i -x_j||=0$, for *i*, *j* in the same ${\mathcal {P}}_{k}$ and $\lim \limits _{t\rightarrow +\infty }||x_i -x_j||\ne 0$, for $i\in {\mathcal {P}}_{k}$ and $j\in {\mathcal {P}}_{l} (k\ne l)$, then it is said that the system (5) achieve a cluster consensus.

According to the definition of matrix-weighted Laplacian *L*, one gets that an equivalent representation of the system (5) is

6 $$\begin{aligned} {\dot{x}}= -Lx, \end{aligned}$$

and multiplicity of zero eigenvalue for the matrix *L* is at least *d* (the details are seen in Ref.^15^). The coordinated behaviors of system (6) can be obtained by the following lemma.

#### **Lemma 1**

*The states of system* (6) *are always convergent. Assumed that multiplicity of zero eigenvalue of the matrix-valued weighted* *Laplacian* *L* *is* *l*, *then there are two conclusions as follows*.

(i) $l=d$ *if and only if the system* (6) *achieves average consensus. Moreover, the states of agents satisfy* $x(t)\rightarrow (\varOmega _1\varOmega _1^T+\varOmega _2\varOmega _2^T+\cdots +\varOmega _d\varOmega _d^T)x(0)$ *as* ${t\rightarrow +\infty }$, *where* $\varOmega _i=\frac{1}{\sqrt{m}}{\mathbf {1}}_m\otimes {\mathbf {e}}_i$ *for* $i=1,2,\ldots ,d.$
(ii) $l>d$ *if and only if this system achieves cluster consensus. The final states of agents are determined by* $(\varOmega _1\varOmega _1^T+\varOmega _2\varOmega _2^T+\cdots +\varOmega _l\varOmega _l^T)x(0)$*, where* $\varOmega _i=\frac{1}{\sqrt{m}}{\mathbf {1}}_m\otimes {\mathbf {e}}_i$ *for* $i=1,2,\ldots ,d$ *and* $\varOmega _i(i=1,\ldots ,l)$ *are pairwise orthonormal eigenvectors associated with zero eigenvalues. In addition, the eigenvectors belonged to* ${\mathcal {N}}(L)\setminus \mathrm {span}\{{{\mathbf {1}}}_m\otimes I_d\}$ *cause cluster consensus of system* (6) *and determine the number of cluster*.

#### *Proof*

Assume that the remaining nonzero eigenvalues of the matrix *L* are $\uplambda _{l+1},\ldots ,$ $\uplambda _{md}$ with $\uplambda _{i}>0(i=l+1,\ldots ,md)$. Note that *L* is semi-positive definite, the matrix *L* is diagonalizable according to Ref.^31^. It directly follows that there exists orthogonal matrix $P=(\varOmega _1,\varOmega _2,\ldots ,\varOmega _{md})$ such that

7 $$\begin{aligned} L=(\varOmega _1,\varOmega _2,\ldots ,\varOmega _{md}) \underbrace{ \left( \begin{array}{cc} \underbrace{\begin{matrix} 0&{} &{} &{} \\ &{}\ddots &{} &{} \\ &{} &{}0&{}\\ \end{matrix}}_l&{} ~\\ ~&{} \begin{matrix} \uplambda _{l+1}&{} &{} &{} \\ &{}\ddots &{} &{} \\ &{} &{}\uplambda _{md}&{}\\ \end{matrix} \end{array} \right) }_\varLambda \left( \begin{array}{c} \varOmega _1^T\\ \varOmega _2^T\\ \vdots \\ \varOmega _{md}^T\\ \end{array}\right) , \end{aligned}$$

where $\varOmega _i$ is eigenvector associated with the *i*th eigenvalue for $i=1,\ldots ,md$ and $\varOmega _i(i=1,\ldots ,l)$ are pairwise orthonormal eigenvectors associated with zero eigenvalues. The solution to ${\dot{x}}=-Lx$ is given by $x(t)=e^{-Lt}x(0)=Pe^{-\varLambda t}P^{-1}x(0)\rightarrow (\varOmega _1\varOmega _1^T+\varOmega _2\varOmega _2^T+\cdots +\varOmega _d\varOmega _d^T+ \varOmega _{d+1}\varOmega _{d+1}^T+\cdots +\varOmega _l\varOmega _l^T)x(0)$ as ${t\rightarrow +\infty }$. Actually, it can be assumed that the first *d* eigenvectors $\varOmega _i\in \mathrm {span}\{{\mathbf {1}}_m\otimes {I}_d\}$ and $\varOmega _i=\frac{1}{\sqrt{m}}{\mathbf {1}}_m\otimes {\mathbf {e}}_i$ for $i=1,2,\ldots ,d$.

(i) For the case that $d=l$, note that the fact $L({\mathbf {1}}_m\otimes I_d)={O}$, where *O* being a zero matrix with appropriate dimension, we can get that $span\{{\mathbf {1}}_m\otimes I_d\}\subset {\mathcal {N}}(L)$. Because the algebraic multiplicity of the zero eigenvalue of matrix *L* is *d*, *L* has at most *d* linearly independent eigenvectors belonged to zero eigenvalues. As a result, ${\mathcal {N}}(L)=span\{{\mathbf {1}}_m\otimes I_d\}$. Thereby, there is $x(t)\rightarrow (\varOmega _1\varOmega _1^T+\varOmega _2\varOmega _2^T+\cdots +\varOmega _d\varOmega _d^T)x(0)$, where
  8 $$\begin{aligned} \varOmega _1\varOmega _1^T+\cdots +\varOmega _d\varOmega _d^T={\mathbf {1}}_m\otimes \left( \begin{array}{c} \frac{1}{m}{\mathbf {1}}_m^T\otimes {\mathbf {e}}_1^T\\ \frac{1}{m}{\mathbf {1}}_m^T\otimes {\mathbf {e}}_2^T\\ \vdots \\ \frac{1}{m}{\mathbf {1}}_m^T\otimes {\mathbf {e}}_d^T\\ \end{array}\right) . \end{aligned}$$
  As a consequence, one can observe that systems achieve average consensus and the consensus state is $\frac{1}{m}({\mathbf {1}}_m^T\otimes I_d)x(0)$, which is in accord with the conclusion in Ref.^31^.
(ii) For the case of $d <l$, there is
  9 $$\begin{aligned} \varOmega _{d+1}\varOmega _{d+1}^T+\varOmega _{d+2}\varOmega _{d+2}^T+\cdots +\varOmega _l\varOmega _l^T= \left( \begin{array}{c} \omega _{d+1}^1\varOmega _{d+1}^T+\omega _{d+2}^1\varOmega _{d+2}^T+\cdots +\omega _{l}^1\varOmega _{l}^T\\ \omega _{d+1}^2\varOmega _{d+1}^T+\omega _{d+2}^2\varOmega _{d+2}^T+\cdots +\omega _{l}^2\varOmega _{l}^T\\ \vdots \\ \omega _{d+1}^{md}\varOmega _{d+1}^T+\omega _{d+2}^{md}\varOmega _{d+2}^T+\cdots +\omega _{l}^{md}\varOmega _{l}^T\\ \end{array}\right) , \end{aligned}$$
  where the vector $\varOmega _{i}=(\omega _{i}^1,\omega _{i}^2,\ldots ,\omega _{i}^{md})^T(i=1,2,\cdots ,l)$. Because $\varOmega _1,\varOmega _2,\ldots ,\varOmega _l$ are pairwise orthogonal, $\varOmega _i^T(i=d+1,\ldots ,l)$ cannot be represented linearly by $\varOmega _1^T,\varOmega _2^T,\ldots ,$ $\varOmega _d^T$^36^, which implies $(\varOmega _{d+1}\varOmega _{d+1}^T+\varOmega _{d+2}\varOmega _{d+2}^T+\cdots +\varOmega _l\varOmega _l^T)x(0)\notin \mathrm {span}\{{\mathbf {1}}_m\otimes I_d\}$. So the final state of solution to the system ${\dot{x}}=-Lx$ can be decompose into
  10 $$\begin{aligned} x(t)\rightarrow \underbrace{(\varOmega _1\varOmega _1^T+\varOmega _2\varOmega _2^T+\cdots +\varOmega _d\varOmega _d^T)x(0)}_{consensus} +\underbrace{( \varOmega _{d+1}\varOmega _{d+1}^T+\cdots +\varOmega _l\varOmega _l^T)x(0)}_{cluster}. \end{aligned}$$
  It is clear that the system ${\dot{x}}=-Lx$ arrives cluster consensus and it can be seen from the structure of final state (10) that the eigenvectors $\varOmega _{d+1},\ldots ,\varOmega _l$ give rise to cluster consensus and the number of cluster is determined up to the eigenvectors $\varOmega _{d+1},\ldots ,\varOmega _l$.

Conversely, if the system ${\dot{x}}=-Lx$ arrive cluster consensus, by virtue of the solution

$$\begin{aligned} x(t)=e^{-Lt}x(0)=Pe^{-\varLambda t}P^{-1}x(0)\rightarrow \underbrace{(\varOmega _1\varOmega _1^T+\varOmega _2\varOmega _2^T+\cdots +\varOmega _d\varOmega _d^T)x(0)}_{consensus}\\ +\underbrace{( \varOmega _{d+1}\varOmega _{d+1}^T+\cdots +\varOmega _l\varOmega _l^T)x(0)}_{cluster}~~({t\rightarrow +\infty }), \end{aligned}$$

which results in $l>d$. The proof are completed. $\square$

#### *Remark 3*

It should be noted that Lemma 1 will play an important role in the sequent design procedure of control law and the essential point is that types of coordinated behaviors for multiple agents have a great relationship with algebraic multiplicity of zero eigenvalues for matrix-weighted Laplacian. According to the above lemma and some conclusions in Ref.^31^, we have *L* only has *d* zero eigenvalues if and only if ${\mathcal {G}}$ can be spanned by a cluster. That is to say, *L* only has *d* zero eigenvalues is equivalent to ${\mathcal {N}}(L)=span\{{\mathbf {1}}_m\otimes I_d\}$. Conservatively, if the networked topology ${\mathcal {G}}$ has a positive spanning tree, matrix-weighted Laplacian *L* only has *d* zero eigenvalues.

To realize coordinated motion for networked Lagrangian agents (1) in the presence of parametric uncertainties, we define the adaptive cooperative control law with $K_i\succ 0$ and $\varUpsilon _i\succ 0$ for the *i*th manipulator as

11 $$\begin{aligned} \left\{ \begin{array}{l}\tau _i= Y_i(q_i,{\dot{q}}_i,q_{r_i},{\dot{q}}_{r_i}){\check{\varTheta }}_i-K_is_i-\sigma \sum \limits \limits _{j=1}^{m}A_{ij}(q_i-q_j),\\ \dot{{\check{\varTheta }}}_i=-\varUpsilon _iY_i(q_i,{\dot{q}}_i,q_{r_i},{\dot{q}}_{r_i})^Ts_i, \end{array} \right. \end{aligned}$$

where ${\check{\varTheta }}_i$ is the estimate of unknown constant vector $\varTheta _i$, and $\sigma =0$ if the matrix-valued weighted Laplacian *L* has just *d* zero eigenvalues, otherwise, $\sigma =1$.

#### *Remark 4*

The adaptive control law (11) is similar to the traditional coordinated protocols^37^ when the matrix-valued weighted Laplacian *L* has just *d* zero eigenvalues. If the matrix *L* has more than *d* zero eigenvalues, additional coupling term $\sum \limits \limits _{j=1}^{m}A_{ij}(q_i-q_j)$ is required to realize the coordination.

Let ${\tilde{\varTheta }}_i=\varTheta _i-{\check{\varTheta }}_i,$ then the dynamics of system (1) with the control protocol (11) can be formulated as

12 $$\begin{aligned} M_i(q_i){\dot{s}}_i+C_i(q_i,{\dot{q}}_i)s _i=-Y_i(q_i,{\dot{q}}_i,{\dot{q}}_{r_i},\ddot{q}_{r_i}){\tilde{\varTheta }}_i-K_is_i-\sigma \sum \limits \limits _{j=1}^{m}A_{ij}(q_i-q_j). \end{aligned}$$

A sliding vector for agent *i* is introduced as follows,

13 $$\begin{aligned} s_i={\dot{q}}_i-{\dot{q}}_{r_i}, \end{aligned}$$

where the reference velocity ${\dot{q}}_{r_i}$ will be designed according to the control target and networked structure.

#### *Remark 5*

The sliding vector $s_i$ in the adaptive control law (13) is, in form, the same as the traditional sliding variable $s_i={\dot{q}}_i-{\dot{q}}_{ri}$ introduced in^32^, and the only difference lies in fact that the structure of virtual reference velocity ${\dot{q}}_{ri}$ is different. More specially, the virtual reference velocity $q_{ri}$ will be designed in sequent sections.

### Stationary coordinated motion

In this subsection, we will consider  stationary coordinated motion of networked Lagrangian agents (1) over matrix-valued weighted networks. The reference velocity ${\dot{q}}_{r_i}$ is given as

14 $$\begin{aligned} {\dot{q}}_{r_i}=-\sum \limits \limits _{j=1}^{m}A_{ij}(q_i-q_j). \end{aligned}$$

Differentiating Eq. (14) along the time *t* can obtain the following acceleration,

15 $$\begin{aligned} \ddot{q}_{r_i}=-\sum \limits \limits _{j=1}^{m}A_{ij}({\dot{q}}_i-{\dot{q}}_j). \end{aligned}$$

Here, the positive semi-definite $A_{ij}$ in the reference velocity (14) or the reference acceleration (15) represents interaction between Lagrangian agent *i* and Lagrangian agent *j*.

To begin with, let $q=(q_1^T,q_2^T,\ldots ,q_m^T)^T, s=(s_1^T,s_2^T,\ldots ,s_m^T)^T,$ $M(q)=$ $\mathrm {blkdiag}$ $(M_1(q_1),$ $M_2(q_2),\ldots ,M_m(q_2)),$ $Y(q,{\dot{q}},{\dot{q}}_{r},\ddot{q}_{r})=\mathrm {blkdiag}(Y_1(q_{r_1},{\dot{q}}_{r_1},$ ${\dot{q}}_{r_1},\ddot{q}_{r_1}), \ldots ,Y_m(q_{r_m},$ ${\dot{q}}_{r_m}, {\dot{q}}_{r_m},\ddot{q}_{r_m}))$, $C(q,{\dot{q}})=\mathrm {blkdiag}(C_1(q_1,\dot{q_1}),$ $C_2(q_2,{\dot{q}}_2),\ldots ,$ $C_m(q_m,{\dot{q}}_m)), {\tilde{\varTheta }}=({\tilde{\varTheta }}_1^T,{\tilde{\varTheta }}_2^T,$ $\ldots ,{\tilde{\varTheta }}_m^T)^T$, and $K=\mathrm {blkdiag}(K_1,K_2,\ldots ,K_m)$, the dynamics (12) is expressed as in the following matrix form:

16 $$\begin{aligned} M(q){\dot{s}}+C(q,{\dot{q}})s=-Y(q,{\dot{q}},{\dot{q}}_{r},\ddot{q}_{r}){\tilde{\varTheta }}-Ks-\sigma Lq. \end{aligned}$$

We consider a Lyapunov-like function

17 $$\begin{aligned} V=\frac{1}{2}\sigma q^TLq+\frac{1}{2} s^TM(q)s+\frac{1}{2}{\tilde{\varTheta }}^T\varUpsilon ^{-1}{\tilde{\varTheta }}, \end{aligned}$$

then the following conclusion will be drawn.

#### **Theorem 1**

*If the matrix-valued weighted Laplacian* *L* *has just* *d* *zero eigenvalues, coupled Lagrangian system* (1) *can achieve complete consensus under the adaptive controller* (11) *with* $\sigma =0$*, otherwise, networked Lagrangian system* (1) *under the controller* (11) *with* $\sigma =1$ *can achieve cluster consensus*.

#### *Proof*

In order to investigate the dynamical behavior of closed-loop system (12), one can obtain the derivative of  *V* along the trajectory of (16) as follows:

18 $$\begin{aligned} \begin{array}{lll} {\dot{V}}&{}=&{}\sigma q^TL{\dot{q}}+ s^TM(q){\dot{s}}+ \frac{1}{2}s^T{\dot{M}}(q)s-{\tilde{\varTheta }}^T\varUpsilon ^{-1}\dot{{\check{\varTheta }}}\\ &{}=&{}\sigma q^TL{\dot{q}}+ s^T(-C(q,{\dot{q}})s-Y(q,{\dot{q}},{\dot{q}}_{r},\ddot{q}_{r}){\tilde{\varTheta }}-Ks-\sigma Lq)+ \frac{1}{2}s^T{\dot{M}}(q)s\\ &{}&{}+{\tilde{\varTheta }}^T Y(q,{\dot{q}},q_{r},{\dot{q}}_{r})^Ts\\ &{}=&{}\sigma q^TL{\dot{q}}- s^TKs-\sigma s^TLq\\ &{}=&{}\sigma q^TL{\dot{q}}- s^TKs-\sigma ({\dot{q}}+Lq)^TLq\\ &{}=&{}- s^TKs-\sigma q^TL^2q. \end{array} \end{aligned}$$

The third equation of (18) is obtained due to the fact that ${\dot{M}}(q)-2C(q,{\dot{q}})$ is skew-symmetric(Property 2).

(i) For the case that the matrix-valued weighted Laplacian *L* has just *d* zero eigenvalues, we introduce a linear transformation as follows: ${\tilde{q}} = {\mathcal {T}}q$, where ${\mathcal {T}}\in R^{md\times md}$ is the transformation matrix defined by

19 $$\begin{aligned} {{\mathcal {T}}}=\left( \begin{array}{ccccc} I_{d} &{} O&{} O&{}\cdots &{} O\\ I_{d} &{}-I_{d}&{}O&{}\ldots &{}O\\ \vdots &{}\vdots &{}\vdots &{}\ddots &{}\vdots \\ I_{d}&{}O&{}O&{}\ldots &{}-I_{d}\\ \end{array}\right) . \end{aligned}$$

It is easy to verify that ${\mathcal {T}}$ is nonsingular and ${\mathcal {T}}^{-1}={\mathcal {T}}$. By linear transformation ${\tilde{q}} = {\mathcal {T}}q=(q_1^T,q_e^T)^T=(q_1^T,(q_1-q_2)^T,\cdots ,(q_1-q_m)^T)^T$, the system ${\dot{q}}={\dot{q}}_r +s=-Lq+s$ can be rewritten as $\dot{{\tilde{q}}}= -{\mathcal {T}}L{\mathcal {T}}{\tilde{q}}+{\tilde{s}}$, where ${\tilde{s}}={\mathcal {T}}s=(s_1^T,(s_2-s_1)^T,\ldots ,(s_m-s_1)^T)^T=(s_1^T,s_e^T)^T$ and

20 $$\begin{aligned} \begin{aligned} \begin{array}{lll} {\mathcal {T}}L{\mathcal {T}}&{}=&{}{\left( \begin{array}{ccccc} \sum \limits _{k=1}^{m}L_{1k}~&{}~ -L_{12}~&{}~ -L_{13}~&{}~ \cdots &{} -L_{1m}\\ \sum \limits _{k=1}^{m}L_{1k}-\sum \limits _{k=1}^{m}L_{2k}~&{}~L_{22}-L_{12}~&{}~ L_{23}-L_{13}&{}\ldots &{}L_{2m}-L_{1m}\\ \vdots ~&{}~\vdots &{}\vdots &{}\ddots &{}\vdots \\ \sum \limits _{k=1}^{m}L_{1k}-\sum \limits _{k=1}^{m}L_{mk}~&{}~L_{m2}-L_{12}~&{}~ L_{m3}-L_{13}&{}\ldots &{}L_{mm}-L_{1m}\\ \end{array}\right) } \\ &{}=&{}\left( \begin{array}{ccccc} O~&{}~ -L_{12}&{} -L_{13}&{}\cdots &{} -L_{1m}\\ O~&{}~ L_{22}-L_{12}~&{}~ L_{23}-L_{13}&{}\ldots &{}L_{2m}-L_{1m}\\ \vdots &{}\vdots &{}\vdots &{}\ddots &{}\vdots \\ O~&{}~ L_{m2}-L_{12}~&{}~ L_{m3}-L_{13}&{}\ldots &{}L_{mm}-L_{1m}\\ \end{array}\right) . \end{array} \end{aligned} \end{aligned}$$

As a matter of fact, the second equation in (20) can be obtained from $\sum \limits _{k=1}^{m}L_{\imath k}=O$ for $\imath =1,2,\cdots ,m.$ Denote ${\mathcal {L}}_{1}=(-L_{12}~~-L_{13}~~\cdots ~~-L_{1m})$, and

21 $$\begin{aligned} {\mathcal {L}}_{e}= \left( \begin{array}{cccc} L_{22}-L_{12}&{}L_{23}-L_{13}&{}\ldots &{}L_{2m}-L_{1m}\\ \vdots &{}\vdots &{}\ddots &{}\vdots \\ L_{m2}-L_{12}&{}L_{m3}-L_{13}&{}\ldots &{}L_{mm}-L_{1m}\\ \end{array}\right) , \end{aligned}$$

one follows that the linear system $\dot{{\tilde{q}}}= -{\mathcal {T}}L{\mathcal {T}}{\tilde{q}}+{\mathcal {T}}s$ can be divided into the following two subsystems

22 $$\begin{aligned} {\dot{q}}_{1}=-{\mathcal {L}}_{1}q_{e}+s_1 \end{aligned}$$

and

23 $$\begin{aligned} {\dot{q}}_{e}=-{\mathcal {L}}_{e}q_{e}+s_e. \end{aligned}$$

Since the matrix *L* has and merely has *d* eigenvalues, all eigenvalues of ${\mathcal {L}}_{e}$ are positive, which implies linear system ${\dot{q}}_{e}=-{\mathcal {L}}_{e}q_{e}$ is exponentially stable. According to the design of control law (11), it can be seen from $V=\frac{1}{2} s^TM(q)s+\frac{1}{2}{\tilde{\varTheta }}^T\varUpsilon ^{-1}{\tilde{\varTheta }}$ and ${\dot{V}}=- s^TKs$ that *V* is monotonically decreasing and has lower bound, which means that $\lim \limits _{t\rightarrow +\infty }V$ exists. Moreover, $s\in L_2\cap L_\infty$ and ${\tilde{\varTheta }}\in \mathrm {L}_\infty$. So there is the result that $s_e\in L_2\cap L_\infty$. For the exponentially stable linear system ${\dot{q}}_{e}=-{\mathcal {L}}_{e}q_{e}+s_e$ with the input $s_e$ and the output $q_{e}$, we have $q_{e}\rightarrow {\mathbf {0}}$ as $t\rightarrow 0$, $q_{e}\in L_2\cap L_\infty$ and ${\dot{q}}_{e}\in L_2$ according to Lemma 2 in Ref.^38^. Therefore, ${\dot{q}}_{r_i}=-\sum \limits \limits _{j=1}^{m}A_{ij}[(q_i-q_1)-(q_j-q_1)]$ is bounded. From the equation ${\dot{q}}=s+{\dot{q}}_r$ together with boundedness of *s*, one can obtain ${\dot{q}}\in L_\infty$, implying that $\ddot{q}_{r}=-L{\dot{q}}\in L_\infty$. Consequently, $Y(q,{\dot{q}},{\dot{q}}_r,\ddot{q}_r)$ is bounded. Due to boundedness of ${\dot{q}}, {\tilde{\varTheta }}, s$, and Property 1, we can deduce that ${\dot{s}}\in L_\infty$ based on the closed-loop system (16). As a consequence, ${\ddot{V}}=- 2s^TK{\dot{s}}\in L_\infty$, which means ${\dot{V}}$ is uniformly continuous. In the light of Barbalats Lemma in Ref.^32^, we draw the conclusion that ${\dot{V}}\rightarrow 0$ when $t\rightarrow \infty$, which lead to the result that $s\rightarrow {\mathbf {0}}$ as $t\rightarrow \infty$. It follows from ${\dot{q}}=s-Lq$ that ${\dot{q}}\rightarrow {\mathbf {0}}$ as $t\rightarrow \infty$.

(ii) If the matrix-valued weighted Laplacian *L* has more than *d* zero eigenvalues, note that $\sigma =1$ according to the control (11), one can conclude from ${\dot{V}}=- s^TKs-q^TL^2q\le 0$ that *V* is monotonically decreasing and has lower bound, which means that $\lim \limits _{t\rightarrow +\infty }V$ exists and *s*, ${\tilde{\varTheta }}$, $q\in L_2\cap L_\infty$. Therefore, it follows from equation (14) that ${\dot{q}}_r\in L_2\cap L_\infty$. Consequently, ${\dot{q}}=s+{\dot{q}}_r \in L_2\cap L_\infty$, giving rise to the boundedness of $\ddot{q}_r$ based on equation (15). According to system (16), we get the result that ${\dot{s}}$ is bounded, which leads to the boundedness of ${\ddot{V}}=- 2s^TK{\dot{s}}-2q^TL^2{\dot{q}}$. As a consequence, ${\dot{V}}$ is uniformly continuous, and we get from Barbalat lemma in Ref.^32^ that ${\dot{V}}\rightarrow 0$ as $t\rightarrow \infty$. This in turn implies that $s\rightarrow {\mathbf {0}}$ and $Lq\rightarrow {\mathbf {0}}$ when $t\rightarrow \infty$, in other word, there are ${\dot{q}}\rightarrow {\mathbf {0}}$ and $\lim \limits _{t\rightarrow \infty }q\in {\mathcal {N}}(L).$ According to Lemma 1, it is straightforward to verify that the generalized position *q* arrive cluster consensus when *L* has more than *d* zero eigenvalues. The proof is completed. $\square$

### Dynamic coordinated motion

This subsection will investigate dynamic coordinated motion for multiple Lagrangian agents (1). To this end, we present the following assumption.

#### **Assumption 1**

All $L_{ii}=D_i=\sum \limits \limits _{j=1}^{m}A_{ij}(i=1,2,\ldots ,m)$ are positive definite.

#### *Remark 6*

Assumption 1 is a standard assumption in the literatures of bearing-based formation control. Actually, if at least two bearings are not collinear, then the bearing Laplacian is a matrix-weighted graph Laplacian and fulfills Assumption 1(The details can be seen in Ref.^39^).

On the basis of Assumption 1, every matrix $L_{ii}(i=1,2,\ldots ,m)$ is invertible. Consequently, the reference velocity ${\dot{q}}_{r_i}$ can be designed as

24 $$\begin{aligned} {\dot{q}}_{r_i}=L_{ii}^{-1}\sum \limits \limits _{j=1}^{m}A_{ij}{\dot{q}}_j-L_{ii}^{-1}\sum \limits \limits _{j=1}^{m}A_{ij}(q_i-q_j). \end{aligned}$$

Evidently, differentiating Eq. (24) along the time *t* can obtain the following acceleration,

25 $$\begin{aligned} \ddot{q}_{r_i}=L_{ii}^{-1}\sum \limits \limits _{j=1}^{m}A_{ij}\ddot{q}_j-L_{ii}^{-1}\sum \limits \limits _{j=1}^{m}A_{ij}({\dot{q}}_i-{\dot{q}}_j). \end{aligned}$$

As a result, the vector form of sliding variable is $s={\dot{q}}-\dot{q_r}=D^{-1}L{\dot{q}}+D^{-1}Lq$, where the block diagonal matrix $D=\mathrm {blkdiag}(L_{11},L_{22},\ldots ,L_{nn})$.

Consider the Lyapunov candidate

26 $$\begin{aligned} V=\frac{1}{2}\sigma q^TLD^{-1}Lq+\frac{1}{2} s^TM(q)s+\frac{1}{2}{\tilde{\varTheta }}^T\varUpsilon ^{-1}{\tilde{\varTheta }}, \end{aligned}$$

now we are in the position to make a statement about dynamic coordinated motion for multiple Lagrangian agents (1).

#### **Theorem 2**

*Under the control adaptive controller* (11) *with the reference velocity* (24), *Lagrangian system* (1) *can achieve complete synchronization if the matrix-valued weighted Laplacian* *L* *just has* *d* *zero eigenvalues, otherwise, the system* (1) *can achieve cluster synchronization*.

#### *Proof*

In order to discuss the dynamical behavior of the closed-loop system (12), one can obtain the derivative of *V* along the trajectory of (12) as follows:

27 $$\begin{aligned} \begin{array}{lll} {\dot{V}}&{}=&{}\sigma q^TLD^{-1}L{\dot{q}}+ s^TM(q){\dot{s}}+ \frac{1}{2}s^T{\dot{M}}(q)s-{\tilde{\varTheta }}^T\varUpsilon ^{-1}\dot{{\check{\varTheta }}}\\ &{}=&{}\sigma q^TLD^{-1}L{\dot{q}}+ s^T(-C(q,{\dot{q}})s-Y(q,{\dot{q}},{\dot{q}}_{r},\ddot{q}_{r}){\tilde{\varTheta }}-Ks-\sigma Lq)+ \frac{1}{2}s^T{\dot{M}}(q)s\\ &{}&{}+{\tilde{\varTheta }}^T Y(q,{\dot{q}},q_{r},{\dot{q}}_{r})^Ts\\ &{}=&{}\sigma q^TLD^{-1}L{\dot{q}}- s^TKs-\sigma s^TLq\\ &{}=&{}\sigma q^TLD^{-1}L{\dot{q}}- s^TKs-\sigma (D^{-1}L{\dot{q}}+D^{-1}Lq)^TLq\\ &{}=&{}- s^TKs-\sigma q^TLD^{-1}Lq. \end{array} \end{aligned}$$

(i) If the matrix-valued weighted Laplacian *L* has just *d* zero eigenvalues, using the linear transformation ${\tilde{q}} = {\mathcal {T}}q$, the system $s=D^{-1}L{\dot{q}}+D^{-1}Lq$ can be rewriting as ${\mathcal {T}}Ds= {\mathcal {T}}L{\mathcal {T}}{\tilde{q}}+ {\mathcal {T}}L{\mathcal {T}}{\tilde{q}}$. Define a new variable $\tilde{{\mathcal {S}}}={\mathcal {T}}Ds=(L_{11}s_1^T,(L_{22}s_2-L_{11}s_1)^T,\ldots ,(L_{mm}s_m-L_{11}s_1)^T)^T =({\mathcal {S}}_1^T,{\mathcal {S}}_e^T)^T$, then

28 $$\begin{aligned} {\mathcal {S}}_1={\mathcal {L}}_1{\dot{q}}_{e}+{\mathcal {L}}_1q_{e}. \end{aligned}$$

and

29 $$\begin{aligned} {\mathcal {S}}_e={\mathcal {L}}_{e}{\dot{q}}_{e}+{\mathcal {L}}_{e}q_{e}, \end{aligned}$$

which implies ${\dot{q}}_{e}=-q_{e}+ {\mathcal {L}}_{e}^{-1}{\mathcal {S}}_e$. Due to $\sigma =0$, there is ${\dot{V}}=- s^TKs.$ The remaining proof involves the same steps as (i) in Theorem 1, it follows that $q_{e}\rightarrow {\mathbf {0}}$ and ${\dot{q}}_{e}\rightarrow {\mathbf {0}}$ as $t\rightarrow \infty$.

(ii) In the case that the matrix-valued weighted Laplacian *L* has more than *d* zero eigenvalues, one can derive that the derivative ${\dot{V}}=- s^TKs-q^TLD^{-1}Lq\le 0$. Similar to the counterpart in the proof of Theorem 1, one can derive that ${\dot{V}}\rightarrow 0(t\rightarrow \infty )$, which means that $s\rightarrow {\mathbf {0}}$ and $Lq\rightarrow {\mathbf {0}}$ due to invertibility of $D^{-1}$. To conclude, there is $\lim \limits _{t\rightarrow \infty }q\in {\mathcal {N}}(L).$ It is obtained by $s=D^{-1}L{\dot{q}}+D^{-1}Lq$ that $\lim \limits _{t\rightarrow \infty }{\dot{q}}\in {\mathcal {N}}(L)$, too. According to Lemma 1, we can verify that *q* and ${\dot{q}}$ arrive cluster synchronization when *L* has more than *d* zero eigenvalues. The proof is completed. $\square$

## Numerical simulations

In order to demonstrate the effectiveness of the presented algorithms, we consider the networked topology containing four agents in $R^2$ and connected weights among agents will be given in sequent examples.

### Single-integrator dynamics

#### Example 1

**Figure 1:**
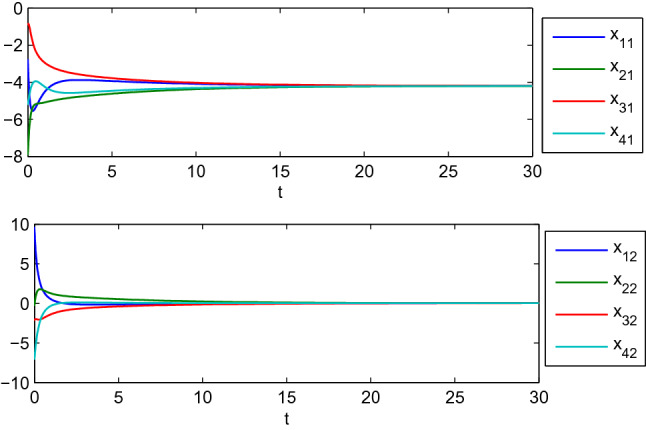
Complete consensus evolution of four single-integrator agents.

**Figure 2:**
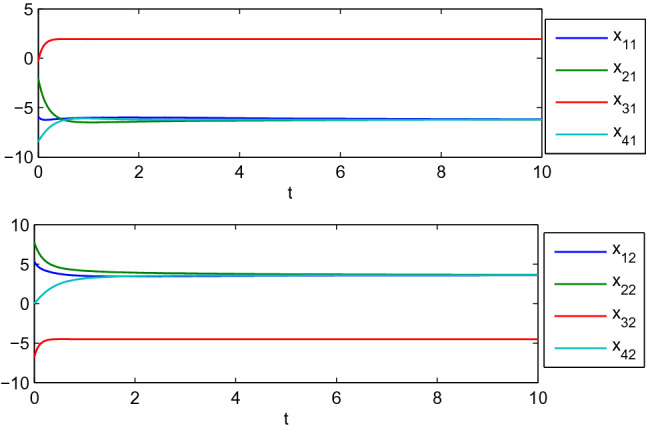
Cluster consensus evolution of four single-integrator agents.

To illustrate Lemma 1, the matrix-weights corresponding to any two single-integrator agents are given by $$A_{12}= \left( \begin{array}{cc} 1&{}1\\ 1&{}1 \end{array} \right) , A_{13}=\left( \begin{array}{cc} 3&{}2\\ 2&{} 2 \end{array} \right) , A_{14}=\left( \begin{array}{cc} 0&{}0\\ 0&{} 1 \end{array} \right) , A_{23}=\left( \begin{array}{cc} 1&{}1\\ 1&{}1 \end{array} \right) , A_{24}=\left( \begin{array}{cc} 1&{}0\\ 0&{}0 \end{array} \right) , A_{34}=\left( \begin{array}{cc} 1&{}1\\ 1&{}1 \end{array} \right) .$$ It is easy to calculate that there are two zero eigenvalues for the matrix-weighted Laplacian *L*. According to Lemma 1, it follows that the position of four agents can reach complete consensus. Simulation results are shown in Fig. 1. However, once matrix-weight $A_{13}$ is switched into $$A_{13}=\left( \begin{array}{cc} 2&{}2\\ 2&{} 2 \end{array} \right)$$, the associated matrix-weighted Laplacian *L* has three zero eigenvalues. One can observe from Fig. 2 that the cluster consensus is reached, which is in accord with Lemma 1.

### Coordinated motion of robotic manipulators

In this subsection, the proposed control algorithms (11) with the reference velocities (14) and (24) are verified by four matrix-weighted coupled two-link manipulators. The details of EL equation (1) are not listed for brevity, but can be found in Ref.^29,40^. The positive definite matrices *K* and $\varUpsilon$ are chosen as $K=20*\mathrm {diag}([1.3,~0.6])\otimes I_4$ and $\varUpsilon =10\otimes I_8$.

#### Example 2

**Figure 3:**
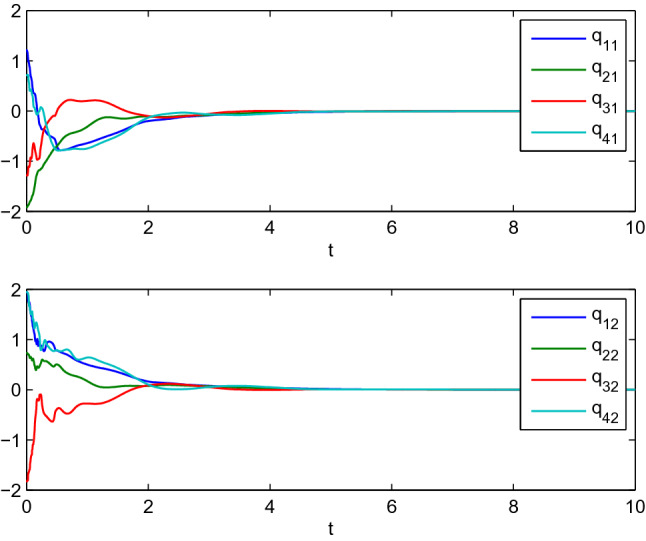
Complete consensus progress for the generalized positions of four two-link manipulators.

**Figure 4:**
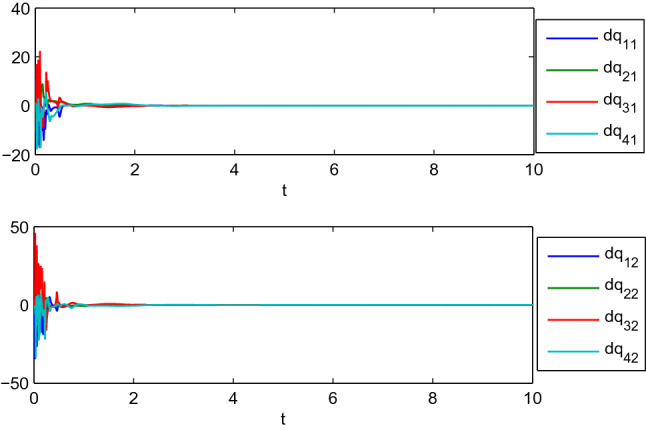
Complete consensus progress for the generalized velocities of four two-link manipulators, where $\mathrm {dq_{i1}}$ and $\mathrm {dq_{i2}}$ represent the 1st and 2nd components of generalized velocities of manipulators $i (i=1,2,3,4)$.

**Figure 5:**
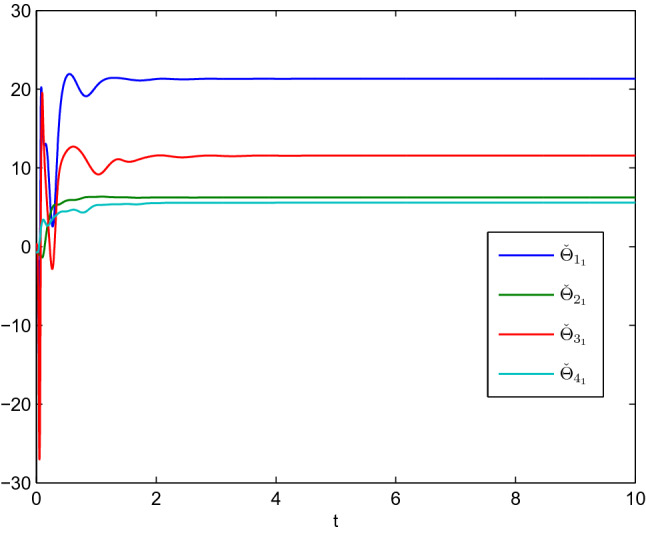
The first uncertain parameters ${\check{\varTheta }}_{i_1}(i=1,2,3,4)$ of all two-link manipulators corresponding to Figs. 3, 4.

**Figure 6:**
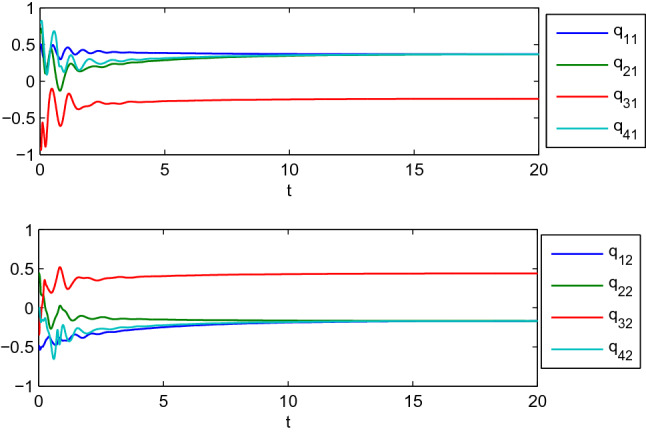
Cluster consensus progress for the generalized positions of four two-link manipulators.

**Figure 7:**
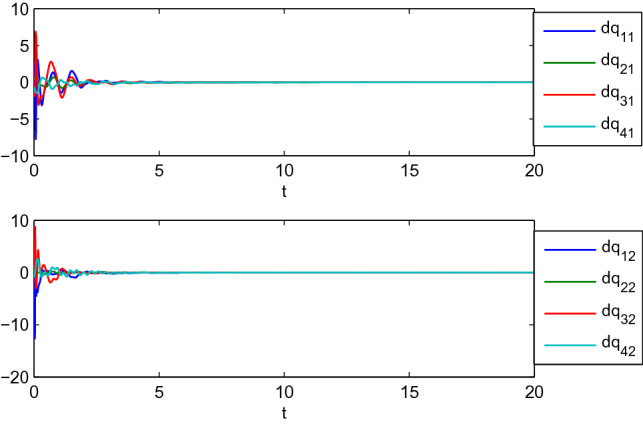
The velocities of four two-link manipulators associated with Fig. 6.

In this example, we consider the stationary motion problem for four manipulators. The matrix-weights are the same as those in the case of complete consensus in Example 1. The initial states, velocities, and uncertain parameters ${\check{\varTheta }}_i$ of the agents are randomly selected in the interval $[-10,10]$. Figures 3, 4 show the positions and velocities of agents 1, 2, 3, and 4 achieve complete consensus and Fig. 5 is the evolution process of the first corresponding parameters of all two-link manipulators. Figure 6 shows the cluster consensus results of generalized positions, and the corresponding velocity states of all the agents are shown in Fig. 7, where the matrix-weights are the same as those in the case of cluster consensus in Example 1.

**Figure 8:**
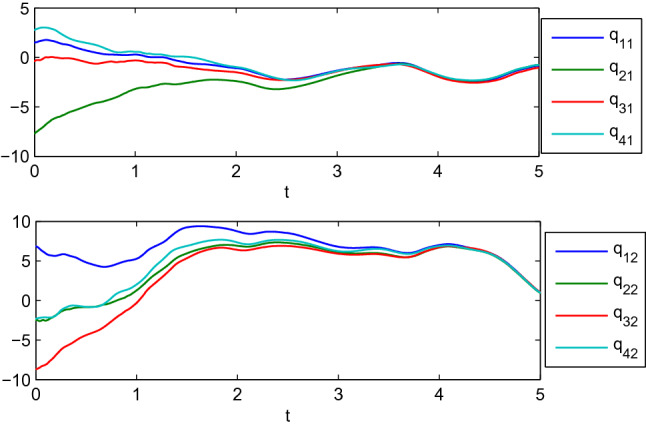
Dynamic coordinated motion for the generalized positions.

**Figure 9:**
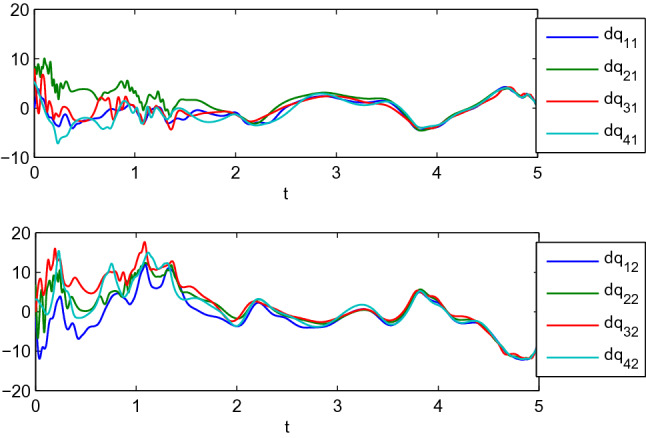
Dynamic coordinated motion for the generalized velocities, where $\mathrm {dq_{i1}}$ and $\mathrm {dq_{i2}}$ represent the 1st and 2nd components of generalized velocities of manipulators $i (i=1,2,3,4)$.

#### Example 3

Let the matrix-weight among four robotic manipulators be, respectively, $$A_{12}= \left( \begin{array}{cc} 1&{}0\\ 0&{}1 \end{array} \right) ,$$ $$A_{13}=\left( \begin{array}{cc} 2&{}0\\ 0&{} 2 \end{array} \right) ,$$ $$A_{14}=\left( \begin{array}{cc} 1&{}0\\ 0&{} 1 \end{array} \right) , A_{23}=\left( \begin{array}{cc} 3&{}1\\ 1&{}3 \end{array} \right) , A_{24}=\left( \begin{array}{cc} 1&{}0\\ 0&{}1 \end{array} \right) , A_{34}=\left( \begin{array}{cc} 2&{}1\\ 1&{}2 \end{array} \right) .$$ We can get there are two zero eigenvalues for the matrix *L* by calculation. Figures 8, 9 show the positions and velocities evolution of four manipulators using the control algorithm (11) with the reference velocities (24) and $\sigma =0$, where the initial values of the four agents are listed in Table 1. It can be observed from Figs. 8, 9 that the four robotic manipulators finally achieve dynamic coordinated motion.

**Table 1:** The initial values of the four robotic agents for dynamic coordinated motion.

	$q_{i_1}$	$q_{i_2}$	${\dot{q}}_{i_1}$	${\dot{q}}_{i_2}$	$\theta _{i_1}$	$\theta _{i_2}$	$\theta _{i_3}$	$\theta _{i_4}$	$\theta _{i_5}$
Agent 1	1.5145	6.8469	− 0.0055	− 1.2195	− 7.0189	− 9.4344	5.1334	5.9221	− 4.1289
Agent 2	− 7.6959	− 2.4982	6.5779	6.8355	3.3048	9.2028	8.8624	− 7.746	2.9657
Agent 3	− 0.3839	− 8.6696	7.9554	− 0.0554	5.4261	− 8.7928	− 4.7509	3.0214	− 7.3279
Agent 4	2.7709	− 2.3011	5.3140	3.0583	− 2.3702	− 3.9996	− 3.1972	8.3785	− 0.8747

## Conclusions

This paper studies coordinated motions of networked robotic manipulators, where interaction weights between agents are characterized by some positive or semipositive definite matrices. Because of the possible existence of positive semi-definite connections, cluster phenomenon naturally occurs in networked system. The research results show that coordinated behaviors of multiple agents are related with the number of zero eigenvalues for Laplacian matrix. On the basis of this, two novel control algorithms are proposed for matrix-weighted networked robotic manipulators such that the agents can reach complete/cluster consensus and complete/cluster synchronization. Several important topics can be developed in the future. For example, we discuss the case where the information interaction between any two manipulators is undirected in this paper, the more complicated case where it is directed should be considered in the next works. In addition, we can discuss an interesting topic that coordinated motions of networked robotic manipulators with both structured uncertainty and unstructured uncertainty.

## Data Availability

The datasets generated during and/or analysed during the current study are available from the corresponding author on reasonable request.
